# A new perspective on endometriosis: Integrating eQTL mendelian randomization with transcriptomics and single-cell data analyses

**DOI:** 10.1007/s10142-025-01543-y

**Published:** 2025-03-26

**Authors:** Sheng Dou, Yi Wei, Zongyun Lin, Hui Wu, Fenglian Yang, Xuechang Cen, Wenjing Lu, Haimei Qin, Rong Wang, Junli Wang

**Affiliations:** 1 The Affiliated Hospital of Youjiang Medical University for Nationalities, Baise, China; 2 Youjiang Medical University for Nationalities, Baise, China; 3 Industrial College of Biomedicine and Health Industry, Youjiang Medical University for Nationalities, Baise, China; 4 Blood transfusion department, Affiliated Hospital of Youjiang Medical University for Nationalities, Baise, China

**Keywords:** Endometriosis, Eutopic, Endometrium, Ectopic, EMT, Ciliated epithelial cells

## Abstract

Endometriosis is caused by the migration of endometrial cells to locations outside the uterine lining. Despite the increasing prevalence of endometriosis, there has been limited research on genetic effects, and its molecular mechanisms remain unclear. This study aimed to investigate the mechanisms underlying the development of endometriosis and to identify new genetic targets for endometriosis by integrating data from gene chips, single-cell mapping, and genome-wide association study databases. Using the Gene Expression Omnibus database, we downloaded data on normal endometrium, eutopic endometrium, and ectopic lesion tissues to explore the differentially expressed genes (DEGs) between normal and eutopic endometrium, and between eutopic and ectopic endometrium. Assessment of the relationships between DEGs and endometriosis involved differential expression, expression quantitative trait loci (eQTL), and Mendelian randomization (MR) analyses. Two single-cell atlas datasets were then analyzed to explore the mechanisms underlying disease development and progression. Intersection of MR results with DEGs between normal and eutopic endometrium highlighted 28 candidate biomarker genes (17 upregulated and 11 downregulated). Similarly, we identified two additional candidate biomarker genes by intersecting the DEGs between eutopic and ectopic endometrium with MR results. Among these 30 candidates, further filtering using single-cell datasets revealed that the histamine N-methyltransferase (*HNMT*), coiled-coil domain containing 28 A (*CCDC28A*), fatty acid desaturase 1 (*FADS1*) and mahogunin ring finger 1 (*MGRN1*) genes were differentially expressed between the normal and eutopic groups, consistent with transcriptomic and MR results. Our findings suggested that eutopic endometrium exhibits epithelial-mesenchymal transition (EMT). Cell communication analysis focused on ciliated epithelial cells expressing *CDH1* and *KRT23* revealed that, in the eutopic endometrium, ciliated epithelial cells are strongly correlated and interact with natural killer cells, T cells, and B cells. We identified four novel biomarker genes and found evidence for EMT in the eutopic endometrium. The mechanism of endometriosis progression may be closely related to EMT and changes in the immune microenvironment triggered by damage to ciliated epithelial cells.

**Supplementary Information:**

The online version contains supplementary material available at 10.1007/s10142-025-01543-y.

## Introduction

Endometriosis is a common gynecological disease, but its diagnosis and treatment pose numerous challenges (Taylor et al. 2021). There is an urgent need to identify efficient biomarkers for the diagnosis and monitoring of progression of this disease (Kiesel and Sourouni 2019, Koninckx et al. 2021). Many studies have screened for biomarkers of endometriosis by selecting genes with differential expression between eutopic endometrium and ectopic lesions (Jiang et al. 2022, Bae et al. 2022, Hosseini et al. 2023, Wang et al. 2022, Wang et al. 2023). However, ectopic lesions and eutopic endometrium are anatomically different, with varying tissue compositions and natural distinctions in gene expression levels of the different tissues and cells types. In my opinion, this method of screening is somewhat flawed and lacks logical consistency. Furthermore, endometriosis is a hereditary disease (Saha et al. 2015). As early as 1980, family studies indicated that first-degree relatives of patients with endometriosis have approximately seven times the risk of the disease compared with the general population (Simpson et al. 1980). In recent years, genome-wide association studies (GWAS) have made significant progress in identifying genetic variants associated with endometriosis (Rahmioglu et al. 2014, Genome-wide 2024). Therefore, the objective of this study was to explore potentially valuable biological targets from multiple angles, including genetics and transcriptomics, by conducting a combined analysis of expression quantitative trait loci (eQTL) data, GWAS data, polygenic risk scores for endometriosis and single-cell atlas data.

The newly identified targets described here were discovered through differential analyses of normal endometrium and eutopic endometrium, with all anatomical locations being situated in the endometrium. This method of target screening was aimed at ensuring rigorous logic. Histamine N-methyltransferase (HNMT) is a key enzyme responsible for the metabolism of histamine. HNMT reduces histamine levels in tissues by converting histamine into N-methylhistamine (Lieberman 2011). In some studies, specific gene polymorphisms of *HNMT *were associated with certain disease susceptibilities (Anvari et al. 2015, García-Martín et al. 2007). However, there have been no reported connections between HNMT and endometriosis in the existing literature. Coiled-coil domain containing 28 A (*CCDC28A*) encodes a protein containing a coiled-coil domain (Zhou et al. 2024). There are no published reports specifically describing the role of CCDC28A in endometriosis. Mahogunin ring finger 1 (*MGRN1*) encodes an E3 ubiquitin ligase with a RING finger domain (Upadhyay et al. 2016). Some studies have reported that MGRN1 is related to tumor cell adhesion and migration (Cerdido et al. 2024), which could make it a valuable new biomarker for the development of endometriosis. Fatty Acid Desaturase 1 (*FADS1*) encodes an enzyme that plays a crucial role in the metabolism of polyunsaturated fatty acids, particularly as a regulator of the synthesis of ω−3 and ω−6fatty acids (Zhao et al. 2020). Although there is no direct link between FADS1 and endometriosis, ω−6 fatty acids are generally considered to promote inflammatory responses, while ω−3fatty acids have anti-inflammatory effects (Simopoulos 2002, Omega-6 2024). Therefore, the function or gene polymorphisms of FADS1 may indirectly influence the development and severity of endometriosis by affecting these metabolic pathways.

**Fig. 1:**
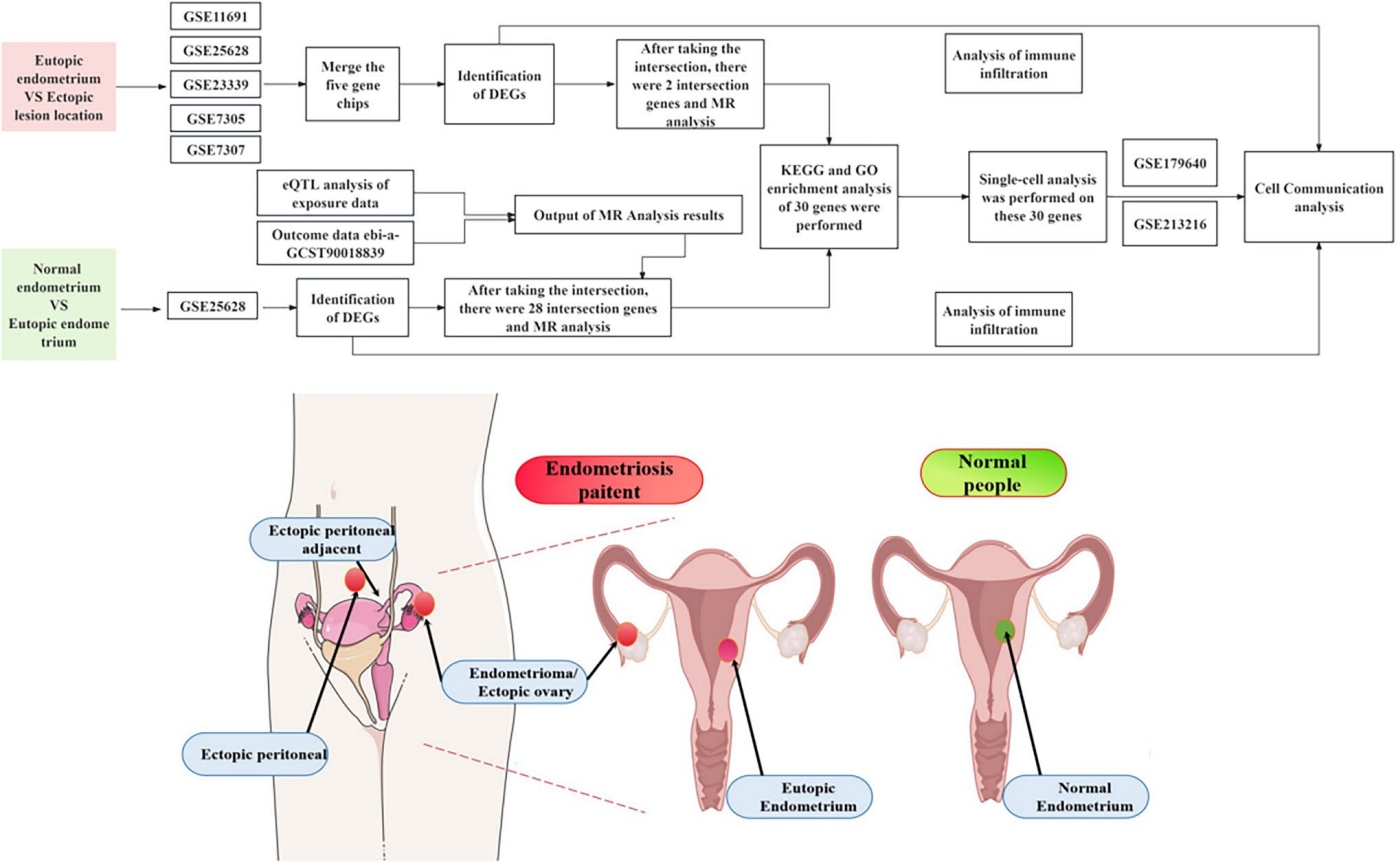
The workflow of analysis

We analyzed changes in the epithelial cell molecular marker CDH1 (Decourtye-Espiard and Guilford 2023, Stehr et al. 2022) between the normal and eutopic groups (GSE120103). Based on the single-cell dataset GSE17964, CDH1 is almost entirely present in epithelial cells. We found that compared to normal endometrium, the proportion of epithelial cells in the eutopic endometrium is significantly reduced. This indicates that EMT has occurred in the eutopic endometrium. Additionally, we examined CDH1 expression in metastatic lesion tissues and the corresponding normal tissues from the same anatomical location.Based on the single-cell dataset GSE213216, CDH1 is almost entirely present in epithelial cells. However, we found that when comparing the peritoneal lesion group and the chocolate cyst lesion group with the normal peritoneum, ovaries, and surrounding fallopian tube regions, there was no change in the proportion of epithelial cells, and the differential expression of CDH1 was not significant. Only the number of inflammatory cells increased. This suggests that significant EMT has not occurred in the ectopic endometrium. Epithelial-mesenchymal transition (EMT) is a cellular biological process in which cells transition from an epithelial phenotype to a mesenchymal phenotype. During this process, epithelial cells lose their intercellular adhesion properties and acquire a more loose, mesenchymal-like phenotype, showing enhanced migratory and invasive capabilities (Owusu-Akyaw et al. 2019). While the occurrence of EMT was obvious in the eutopic endometrium, it was not detected in the lesion group data presented here, and requires further consideration. Activation of the EMT may promote the migration and implantation of endometrial cells in vitro, thus driving the progression of endometriosis. Furthermore, this study found that CDH1 was primarily expressed in ciliated epithelial cells. Subsequent cell communication analysis on ciliated epithelial cells, combined with transcriptomic immune infiltration analysis, revealed a close interaction between ciliated epithelial cells and natural killer (NK) cells in certain receptor-ligand pathways that is explained in the Discussion section. In summary, through eQTL Mendelian randomization (MR) combined with transcriptomic and single-cell analysis, new insights into the development of endometriosis and potential biomarkers can be explored. The workflow of analyses conducted in this study is shown in Fig.1.

## Materials and methods

### Dataset downloading and merging

We downloaded the dataset GSE25628 from the Gene Expression Omnibus (GEO) database (https://www.ncbi.nlm.nih.gov/geo/) to investigate the differentially expressed genes (DEGs) between normal endometrium and ectopic endometrium. We also downloaded datasets GSE11691, GSE23339, GSE25628, GSE7305, and GSE7307 to obtain the probe matrix file, platform file, and clinical information file for each. Using the annotation data from the platform file, we established a map of probes and genes. Subsequently, we used a Perl script to convert the probe matrix into a gene expression matrix. These five datasets were also combined, employing principal component analysis (PCA) to correct for batch effects, with the aim of exploring the DEGs between ectopic and normal endometrium using a large amount of data. Additionally, we downloaded the single-cell datasets GSE213216 and GSE179640 for further analysis. All sample information used in this study is provided in Supplementary Table 1.

### eQTL analysis of exposure data

To identify genetic variations associated with gene expression levels, we conducted an eQTL analysis using transcriptome and genotype data from different cohorts. In 2013, Westra et al. conducted the most extensive meta-analysis of eQTL data to date, including peripheral blood eQTL data from 5,311 individuals from Europe (Westra et al. 2013). The eQTL data used in this study were obtained from the GWAS Catalog website (https://gwas.mrcieu.ac.uk/*).* Using the R package TwoSampleMR, we identified strongly associated single-nucleotide polymorphisms (SNPs, *P* < 5e-08) as instrumental variables (IVs). The linkage disequilibrium parameters were set to R^2^ < 0.001 and clumping distance = 10,000 kb. We excluded SNPs with weak associations or insufficient explanation of phenotypic variance, applying an “F-test Value > 10” filter.

### Determination of outcome data

Summary outcome data were sourced from the genetic association database available in the GWAS Catalog (https://gwas.mrcieu.ac.uk/), using a GWAS ID (ebi-a-GCST90018839) that includes 4,511 endometriosis cases from patients of European ancestry and 231,771 controls, encompassing 24,089,752 SNPs.

### MR analysis

We performed MR analysis using the R package TwoSampleMR. The inverse variance-weighted (IVW) method was used to study relationships between endometriosis and specific genes. Additionally, we conducted further sensitivity analyses using MR-Egger, simple mode, weighted median, and weighted mode methodologies (Birney 2022, Dudbridge 2021). Disease-related genes were identified in three steps: (1) initial selection of genes with a*P* value < 0.05 using the IVW method; (2) refinement of the genes based on the consistency of the direction of MR results (odds ratio [OR] values) across the three different methods; and (3) exclusion of genes showing pleiotropic effects and with a *P* value < 0.05. Next, we intersected the MR-identified endometriosis-related genes with the DEGs between eutopic and normal endometrium, including both upregulated and downregulated genes. Similarly, we intersected endometriosis-related genes with the DEGs between eutopic and ectopic endometrium. Subsequently, all intersecting genes were subjected to MR analysis to determine their causal relationship with the disease. This analysis included heterogeneity tests, pleiotropy tests, and leave-one-out sensitivity analyses to assess the robustness and reliability of the results. Scatter plots, forest plots, and funnel plots were employed to visually present the findings.

Reliable MR analysis is based on three core assumptions: (1) the relevance assumption (the IV is strongly associated with the exposure, but not directly related to the outcome); (2) the independence assumption (the IV is not associated with confounding factors); and (3) the exclusion restriction assumption (the IV affects the outcome only through the exposure; if the IV affects the outcome through other pathways, it is considered to exhibit pleiotropy). In this analysis, R language (version 4.2) was used for all computations. All statistical tests were two-sided, with a *P* value < 0.05 considered to indicate statistical significance.

### Single-cell data analysis

We analyzed samples derived from normal endometrium, eutopic endometrium, and ectopic lesions. After merging the data from each group into a matrix, we used Harmonity algorithm (version 1.0) for batch correction to reduce errors and enhance cell clustering (Korsunsky et al. 2019). Next, we performed dimensionality reduction using Uniform Manifold Approximation and Projection (UMAP). Clustering analysis was conducted using the Leiden community detection algorithm (Becht et al. 2018, Traag et al. 2019). We calculated the median distance from cells to the centers of their respective clusters. Finally, we annotated the cells based on the original publication of dataset GSE179640. Save as above, we analyzed GSE213216 using the same methods.

### Cell communication analysis

We extracted the normal and eutopic endometrial sample groups from the GSE179640 dataset for combined analysis. Using R packages such as NMF, ggplot2, ggalluvial, svglite, and CellChat, we performed cell communication analyses of normal and eutopic endometrium, filtering out cell communications involving < 10 cells. Next, we further analyzed cell communication in ciliated epithelial cells. We inferred intercellular communication at the signaling pathway level, deduced interaction networks at the pathway level, and summarized and integrated the computational results to present the overall cell communication status.

### Clinical sample validation

Clinical samples were collected from patients of the Affiliated Hospital of Youjiang Medical University for Nationalities. All procedures were approved by the ethics committees of the Affiliated Hospital of Youjiang Medical College for Nationalities. Ethical approval number: 2,024,042,302. The approval date is April 23, 2024. Normal endometrium from patients undergoing total hysterectomy not due to endometriosis and ectopic lesion tissues from endometriosis patients were collected and rapidly placed in liquid nitrogen. Before processing, the samples were quickly thawed. TRIzol reagent was added (1 mL/170 mg tissue), and the samples were ground in liquid nitrogen to ensure full contact with the reagent. After centrifugation at 12,000 rpm for 13 min, the samples were left on ice for 20 min. Chloroform was added, shaken to mix, and the RNA-containing supernatant was transferred to a new centrifuge tube. An equal volume of isopropanol was added to the supernatant, mixed well, and then 75% ethanol was added. After thorough mixing, the RNA was precipitated by centrifugation. The RNA pellet was washed twice with ethanol and air-dried. The RNA was then reverse transcribed using a reverse transcription kit (R333-01; Vazyme) with SYBR Green dye (11201ES08; Hieff). The change in fluorescence signal was monitored in real-time, and the threshold cycle (Ct) value was recorded.

### Immune infiltration analysis

After correcting and merging the chip data, we performed comparative immune infiltration analyses between the normal and in eutopic groups, and between the in eutopic and ectopic lesion groups. The CIBERSORT algorithm was used to calculate the relative proportions of immune cell types in each sample, using the R packages BiocManager and preprocessCore, with the following parameters: permutations = 1000 and *P* < 0.05. Further immune-related analyses were conducted using the limma, dplyr, tidyverse, ggplot2, reshape2, ggpubr, and corrplot R packages. Finally, correlation plots and bar charts of related genes, including *CDH1* and *KRT23*, were obtained.

## Results

### Transcriptome data analysis of normal, eutopic, and ectopic endometrial samples

**Fig. 2:**
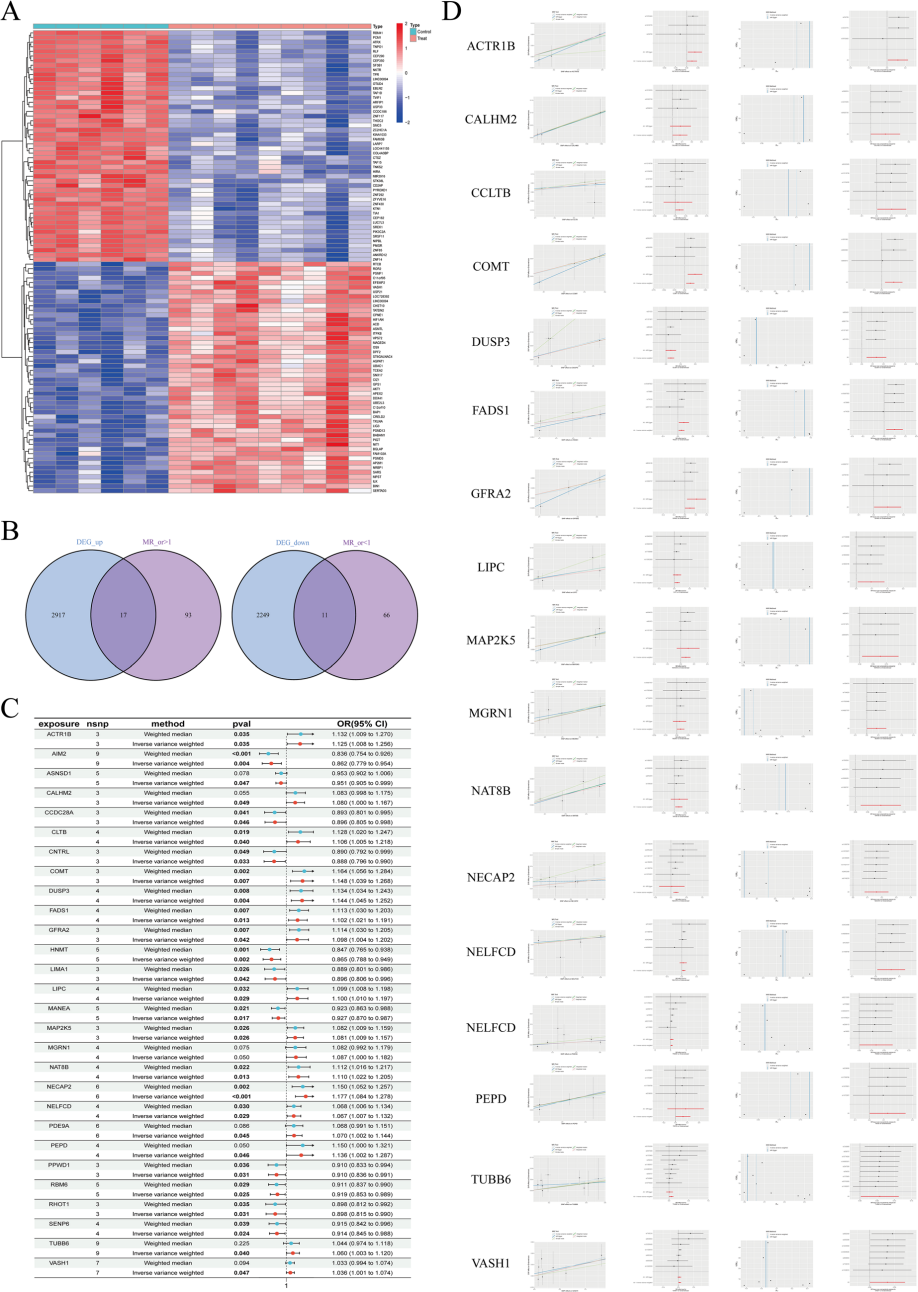
**A**: Extract data from the normal group and eutopic group in the GSE25628 dataset for differential analysis and draw a heatmap. **B**: Intersect the downregulated genes in the transcriptome with the low-risk genes identified by Mendelian randomization. Similarly, intersect the upregulated genes with the high-risk genes. **C**: Plot a forest plot for the 28 intersecting genes. If the OR value is greater than 1, the gene is considered a high-risk gene, indicating that as the expression of this gene increases, the incidence of the disease also increases. If the OR value is less than 1, it is considered a low-risk gene, and as the expression of this gene increases, the incidence of the disease decreases. **D**: Visualize the results for the 17 genes identified as high-risk (OR > 1) through Mendelian randomization using scatter plots, forest plots, funnel plots, and leave-one-out sensitivity analysis. Scatter plot: The horizontal axis represents the effect of SNPs on the gene (exposure factor), and the vertical axis represents the effect of SNPs on endometriosis (outcome). The points represent SNPs, the white horizontal lines represent the range of SNP fluctuations on the gene, and the vertical lines represent the range of SNP fluctuations on endometriosis. Forest plot: The horizontal axis represents the effect size of each SNP (instrumental variable) on the outcome, and the vertical axis represents the SNP. When the effect size is greater than 0, the SNP is considered a risk factor. When the effect size is less than 0, the SNP is considered a protective factor. Funnel plot: If the SNPs are symmetrically distributed on both sides in the inverse variance weighted method, the data are considered to have no significant heterogeneity. Leave-one-out sensitivity analysis: In this analysis, one SNP is removed at a time, and Mendelian randomization is performed using the remaining SNPs

Analysis of dataset GSE25628 showed upregulation of 2934 genes and downregulation of 2260 genes in eutopic endometrium compared with that in normal endometrium (Fig. 2A, B). To compare eutopic and ectopic endometriosis, (Fig. 4) we combined five datasets and employed PCA to eliminate batch effects **(**Fig. 4A**)**. We identified 362 upregulated genes and 276 downregulated genes (Fig. 4B, C, D).

**Fig. 3:**
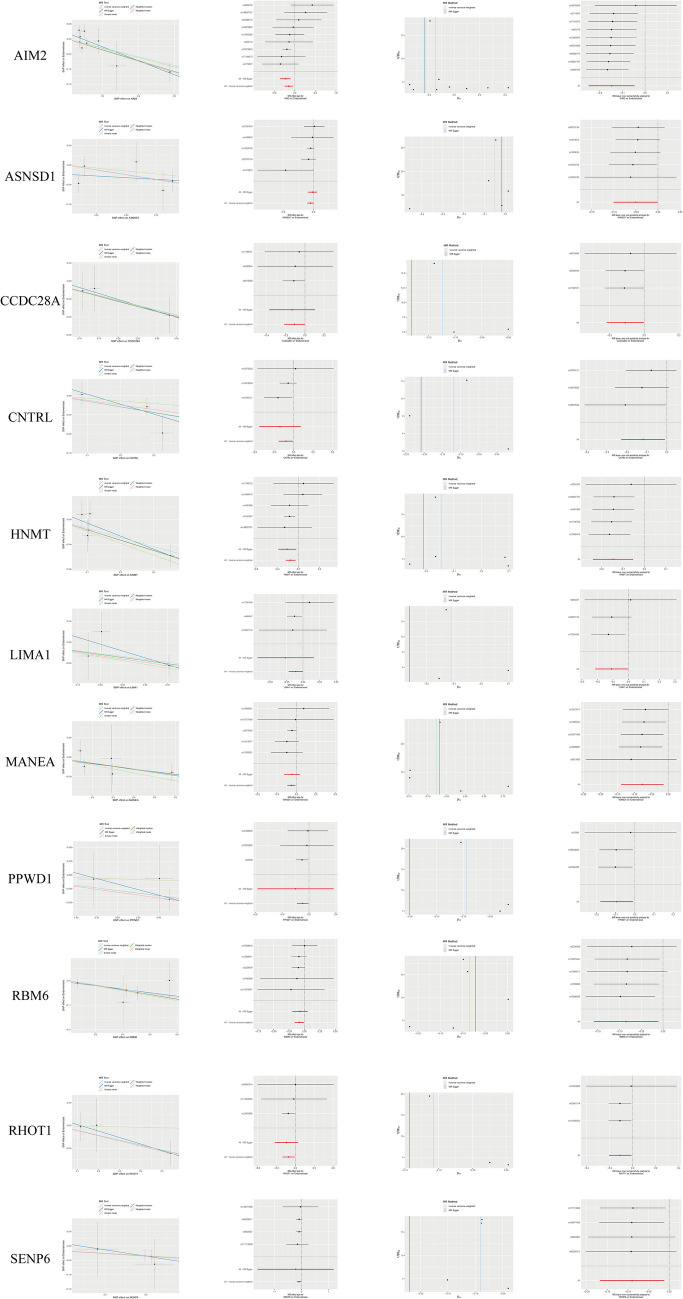
Visualize the results for the 11 genes identified as low-risk (OR < 1) through Mendelian randomization using scatter plots, forest plots, funnel plots, and leave-one-out sensitivity analysis

**Fig. 4:**
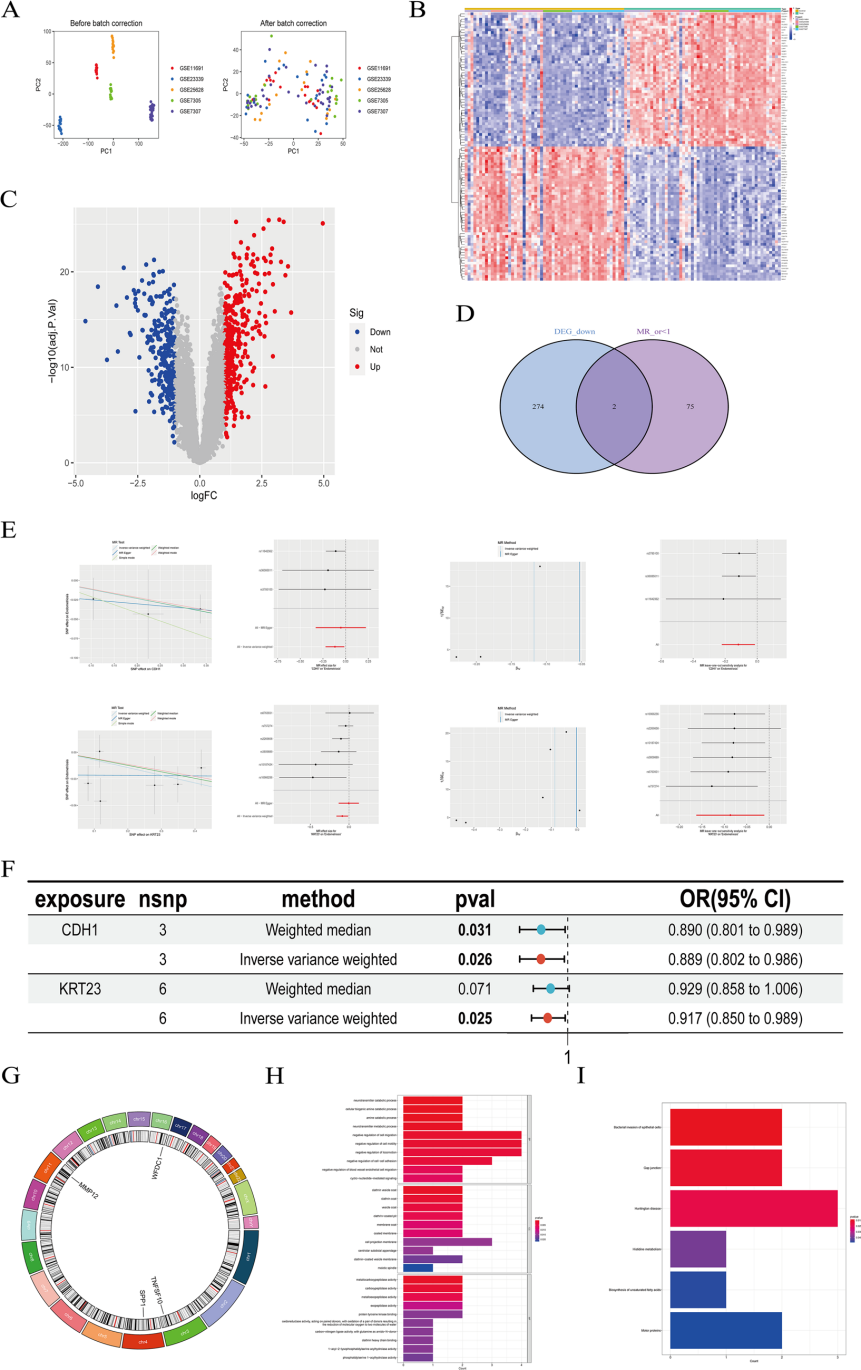
Combine the gene chips from five datasets and perform PCA correction. **B**,** C**: Compare and perform differential analysis on the eutopic endometrium group and ectopic endometrium group within the datasets, then create a heatmap and a volcano plot, respectively. **D**: Intersect the differentially expressed genes with the risk genes identified through Mendelian randomization, resulting in two low-risk genes. This indicates that as the expression levels of the CDH1 and KRT23 genes decrease, the incidence of the disease increases. Conversely, as the expression of these two genes decreases, the likelihood of disease onset decreases. **E**,** F**: Visualize the results for the 11 genes identified as low-risk (OR < 1) through Mendelian randomization using scatter plots, forest plots, funnel plots, and leave-one-out sensitivity analysis. **G**: Display the chromosomal location distribution of 30 selected genes. **H**: Perform GO enrichment analysis on the 30 selected genes. **I**: Perform KEGG enrichment analysis on the 30 selected genes

### MR analysis

Extracted exposure factors were saved in the file named (Supplementary exposure_data.csv). After filtering with F-test > 10, we obtained 26,125 SNPs as strongly associated IVs, with the results saved in (Supplementary exposure.F.csv). The SNPs used as Instrumental variables for MR were saved in (Supplementary table.SNP.csv). The results of MR analysis using five different methods (MR Egger, Weighted median, Inverse variance weighted, Simple mode, Weighted mode) were saved in (Supplementary table.MRresult.csv). Among these methods, if the *P* value using the IVW method was < 0.05, we considered there to be a causal relationship between the exposure factor and the risk of endometriosis. If the β value was < 0, the exposure factor was considered a protective factor, and if the β value was > 0, the exposure factor was considered a risk factor. If the OR was > 1, the exposure factor was considered a risk factor; i.e., as the gene expression level increases, the risk of developing endometriosis also increases. Conversely, if the OR was < 1, the exposure factor was considered a protective factor; i.e., as the gene expression level increases, the risk of developing endometriosis decreases. Next, we used two methods to test for heterogeneity. When the heterogeneity test *P* value was > 0.05, we considered there to be no heterogeneity in the data, and the results were saved in (Supplementary table.heterogeneity.csv). Next, we further filtered and selected genes as follows: (1) genes for which the analysis results were consistent across all five methods were selected; and (2) genes with a pleiotropy test *P* value < 0.05 were excluded. The results were saved in (Supplementary IVW.filter.csv). Finally, we intersected these genes with the DEGs.Intersection of the DEGs between normal and eutopic endometrium identified 17 upregulated genes (Fig. 2D) and 11 downregulated genes (Fig. 3). To visualize the MR results of these 28 genes we used forest plots (Fig. 2C), and illustrated the findings in further detail using scatter plots, forest plots, and funnel plots **(**Figs. 2D, 3 and 4E**)**. *CDH1* and *KRT23* were the only two DEGs at the intersection between eutopic and ectopic endometrium (Fig. 4D), and we created a forest plot for the genes (Fig. 4F). We observed that, in all five algorithms, the expression levels of *CDH1* and *KRT23* were inversely related to the risk of developing endometriosis. In other words, the lower the expression of these two genes, the higher the risk of endometriosis. Finally, we visualized the chromosomal locations of these 30 intersecting DEGs **(**Fig. 4G**)**.

### Gene ontology (GO) and kyoto encyclopedia of genes and genomes (KEGG) enrichment analyses

We conducted KEGG and GO enrichment analyses of these 30 DEGs **(**Fig. 4H, I**)**. The GO enrichment analysis revealed significant enrichment for processes related to the negative regulation of cell motility and migration, as well as the negative regulation of endothelial cell proliferation. The KEGG enrichment analysis showed that these DEGs were enriched in pathways involving bacterial invasion of epithelial cells and gap junctions. At this point, we strongly suspected that these 30 DEGs may have potentially unexplored roles in the migration of endometriotic cells, including their ectopic presence in other locations, providing a wellspring for future research directions. Additionally, we observed enrichment in histidine metabolism, which could support researchers studying the metabolomics of endometriosis.

### Single-cell dataset analysis

**Fig. 5:**
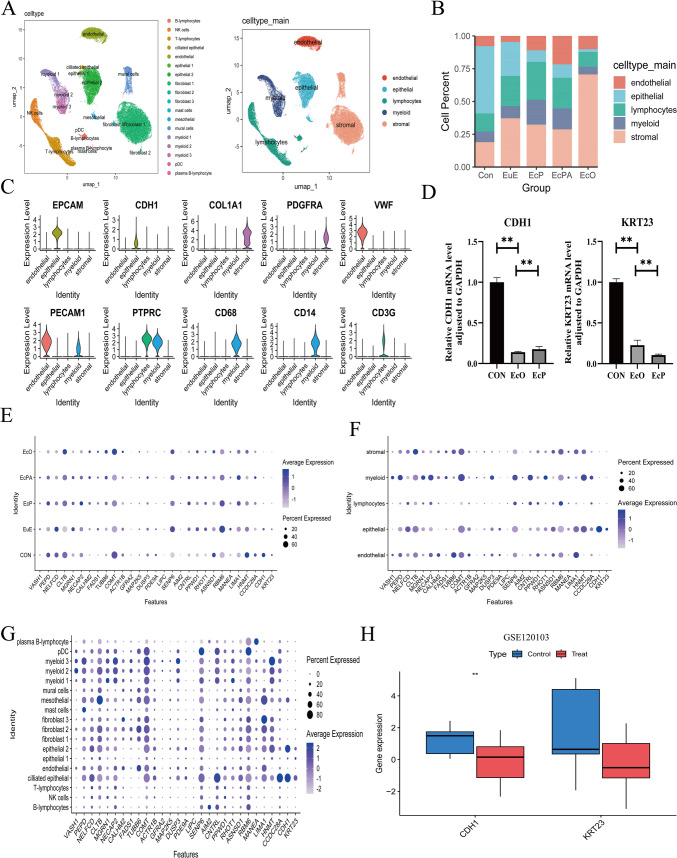
**A** Analyze the GSE179640 dataset, perform dimensionality reduction using UMAP, and conduct a detailed classification **B**: Display the proportions of cells in different groups.of the cell populations. **C**: Annotate the cell markers. **D**: Validate the differential expression of CDH1 and KRT23 in normal tissues and ectopic lesion tissues (Ovarian lesion, peritoneal lesion) using qPCR. **E**,** F**,** G**: Show the detailed differential expression of the 30 selected genes across different groups and various cells. **H**: Use the GSE120103 dataset to further verify whether there is a difference in the expression levels of CDH1 and KRT23 between normal endometrium and eutopic endometrium

We extracted the following from dataset GSE179640: three normal endometrium control (CON) samples, nine eutopic endometrium (EuE) samples, three ectopic ovary (EcO) samples, eight ectopic peritoneal (EcP) samples, and six ectopic peritoneal adjacent (EcPA) samples. After combining these samples, we performed an analysis using UMAP for dimensionality reduction and clustering. Based on molecular markers from the published article associated with the dataset (PMID: 35864314), we manually annotated five cell types: endothelial, epithelial, lymphocytes, myeloid, and stromal cells (Fig. 5A).

Compared with the normal group, we found that the number of epithelial cells was lower while the number of stromal cells was higher in the eutopic group. Because the normal and eutopic endometrium samples were collected from the same anatomical location, the between-group differences identified in subsequent single-cell analyses are crucially important. And the analysis between the normal and eutopic groups is logical. CDH1 is a well-known molecular marker for endothelial cells (Lamouille et al. 2014, Dongre and Weinberg 2019) (Fig.5C). Further analysis revealed that CDH1 expression levels were higher in the normal group than in the eutopic group (Fig. 7B). Fortunately, using transcriptome data from the validation set GSE120103, we also confirmed that the levels of CDH1 were higher in normal endometrium than in eutopic endometrium, whereas KRT23 levels showed no statistical differences. Through quantitative (q)PCR experiments using clinical samples from normal endometrium and eutopic endometrium, and ultimately ectopic endometrium, we validated that the expression levels of *CDH1* and *KRT23* significantly decreased (Fig. 5D). Next, we analyzed the normal endometrium and the eutopic endometrium. Single-cell analysis of the 30 DEGs obtained through the combination of transcriptomics and MR was used to identify four new biomarkers: *HNMT*, *CCDC28A*, *MGRN1* and *FADS1*. These biomarkers not only showed statistically significant differences in transcriptomics analysis, but also exhibited distinct differences in single-cell data. Most importantly, the gene expression trends of these four biomarkers were consistent with the MR results. Because normal and ectopic endometrial samples are derived from the same anatomical location, we consider these biomarker genes to hold great research value.

Compared with the normal and eutopic endometrium groups, the expression levels of *CDH1* and *KRT23* were significantly decreased in the ectopic lesion group (Fig. 5D, E). Additionally, compared with the normal group, the eutopic group showed a decrease in epithelial cells and an increase in stromal cells, leading us to conclude that EMT had occurred in the eutopic endometrium (Fig. 5B).

Many studies suggest that a decrease in epithelial cells and an increase in stromal cells mark the occurrence of EMT (Owusu-Akyaw et al. 2019, Kusama et al. 2021). However, upon closer examination, we must consider that the anatomical location and cellular composition of eutopic endometrial tissues are different from those of ectopic lesions in the ovary or peritoneum. Specifically, if the proportion of epithelial cells in normal peritoneal tissue and surrounding ovarian tissue is indeed lower than that in endometrial tissue, it might be misleading to assume that a decrease in*CDH1* expression in ectopic lesions indicates EMT.

**Fig. 6:**
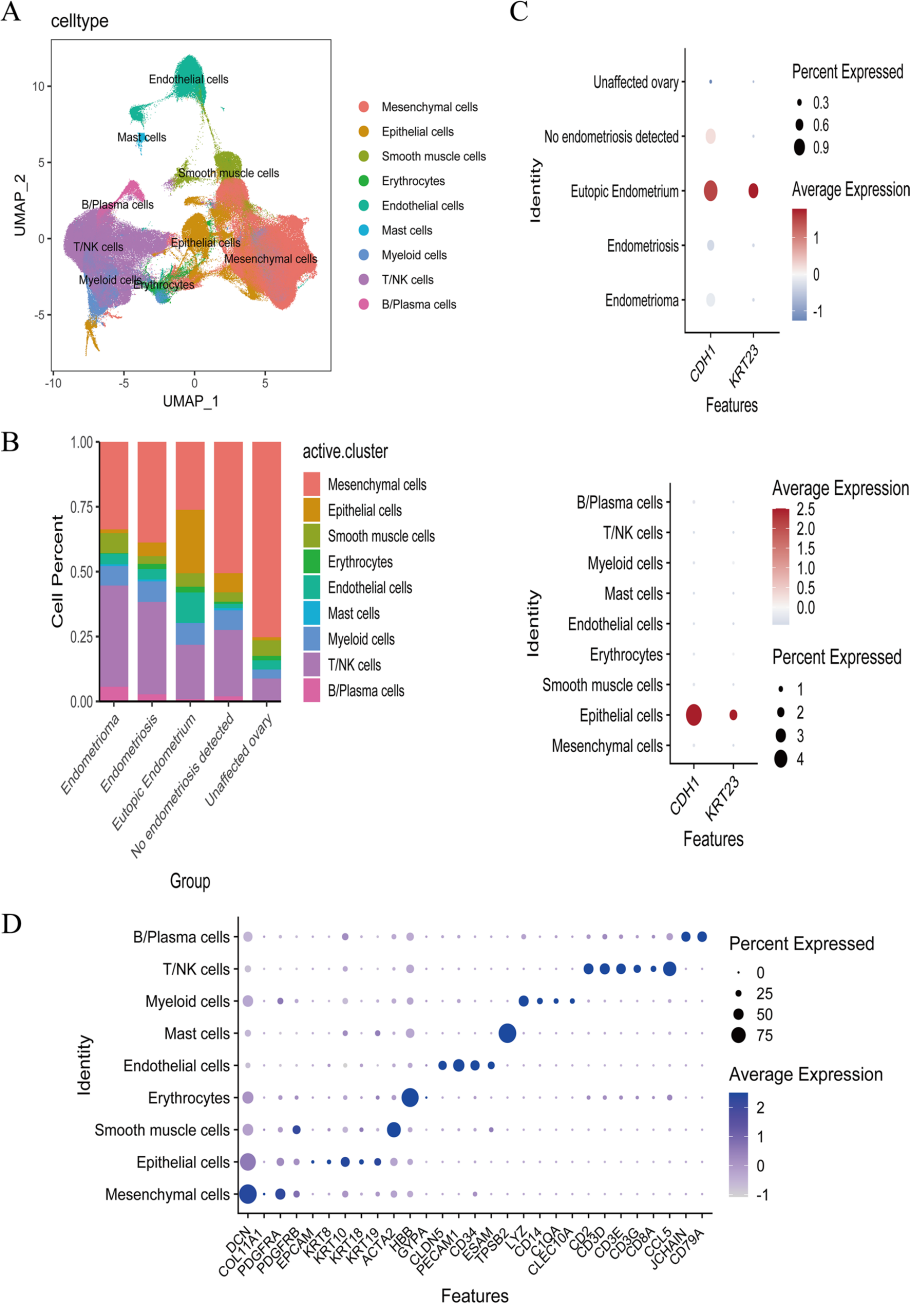
**A**: Analyze the GSE213216 dataset, perform dimensionality reduction using UMAP. **B**: Create a bar chart showing the proportions of different cell types in various groups. **C**: Display the cell annotation information. **D**: Visualize the expression levels of CDH1 and KRT23 across different groups and within different cell types

To address this confusion, we analyzed GSE213216, another comprehensive single-cell transcriptomic analysis of endometriosis with data grouping that could help to resolve the uncertainties raised from our analysis of the previous dataset. We noticed that there were no significant differences in the expression levels of *CDH1* and *KRT23* between the endometrioma and unaffected ovary groups in this dataset (Fig. 6C). However, there were significant differences in cellular composition between these two groups. Compared with the unaffected ovary group, the endometrioma group was heavily infiltrated with inflammatory cells, including substantial increases in B cells and T cells (Fig. 6B). Therefore, we believe that the ectopic group only had a large number of inflammatory cell infiltration, but not that EMT occurred. Finally, we consider this comparison between diseased and non-diseased tissues from the same anatomical location to be reasonable and meaningful.

Many previous studies chose instead to compare eutopic endometrium with ectopic tissues, concluding that EMT had occurred in the ectopic endometrium (Wang et al. 2023, Zhou et al. 2023, Ji et al. 2022). The different anatomical locations of these tissues lead to inherent differences in cellular compositions and proportions of cell types, calling into question the logic of such an approach. Furthermore, this oversight could lead to inaccurate conclusions in differential analyses of transcriptomic and single-cell data. In many articles, the conclusion that*CDH1* levels drop sharply in ectopic lesions and that EMT occurs in ectopic lesions, including ovary lesions, is questionable and requires further consideration. Based on current evidence, the occurrence of EMT in eutopic endometrium is indisputable. However, whether EMT occurs in ectopic lesions is a matter that needs careful examination.

**Fig. 7:**
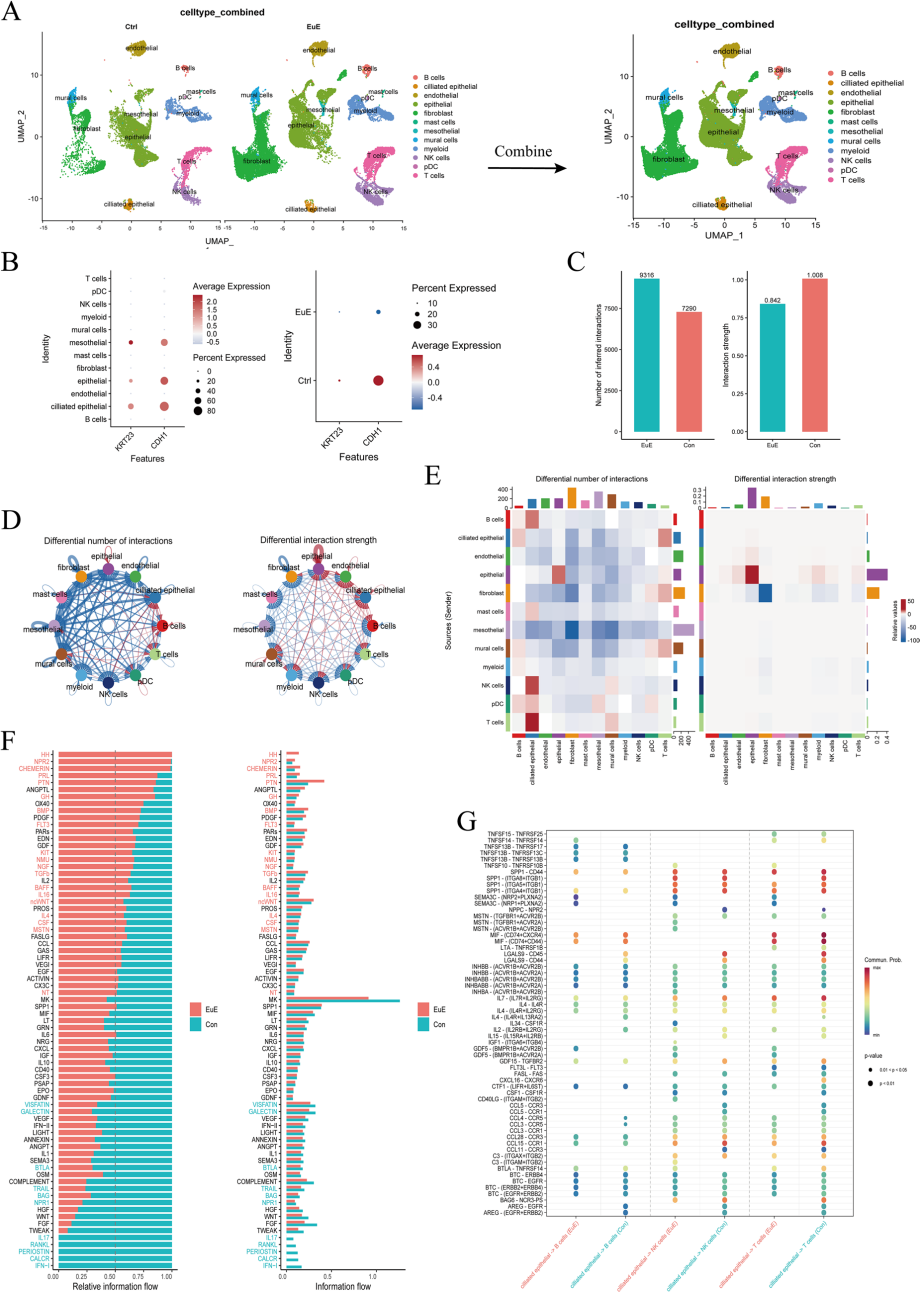
**A** Extract and merge the data from the normal group and eutopic group in the GSE179640 dataset. **B**: Perform a separate analysis of KRT23 and CDH1. **C**: Conduct cell communication analysis between the two groups, focusing on overall cell interaction strength and the number of interactions. **D**,** E**: Visualize the number and strength of cell interactions using circos plots and heatmaps. Red indicates increased cell interactions in the eutopic group, while blue indicates a decrease. We found that compared to the normal group, interactions between ciliated epithelial cells and T cells, B cells, and NK cells are enhanced in the eutopic group. **F**: Visualize the pathway strength differences between the normal and eutopic groups. **G**: Display the changes in receptor-ligand interactions between ciliated epithelial cells and B cells, T cells, and NK cells in the normal and eutopic groups

Additionally, we extracted and merged data from the eutopic and normal endometrium datasets for cell communication analysis (Fig. 7A). We found that *CDH1* and *KRT23* are primarily expressed in ciliated epithelial cells (Fig. 5G). In the endometrium, ciliated epithelial cells are mainly located in the fallopian tubes. These cells play crucial roles in moving eggs and embryos, which are essential for successful conception (Kusama et al. 2021, Lyons et al. 2006). The ciliary motion helps clear secretions and small particles from the fallopian tubes and endometrium, maintaining the patency of the fallopian tubes. Next, we further analyzed the ciliated epithelial cells to visualize the strength of their interactions with other cell types (Fig.7D, E). We found that, compared with normal endometrial tissue, the interactions of ciliated epithelial cells with T cells, B cells, and NK cells were enhanced in the eutopic endometrium. This suggested that ciliated epithelial cells in the eutopic endometrium influence the immune environment. To verify this, we performed immune infiltration analyses of the transcriptome data between the normal and eutopic groups, and between the eutopic and ectopic groups (Figure S1). Compared with the normal group, there was a statistically significant increase in the number of activated NK cells in the eutopic group (*P* < 0.05) (Figure S1E). Next, we performed receptor-ligand interaction analyses of ciliated epithelial cells with NK cells, T cells, and B cells in the normal and eutopic groups (Fig. 7G). This revealed significant enhancement of the interactions between ciliated epithelial cells and NK cells, mediated through the transforming growth factor (TGF)-β and tumor necrosis factor (TNF) signaling pathways, in the eutopic group. Additionally, there was an increase in the involvement of pathways involving cytokine-cytokine receptor interaction and viral protein interaction with cytokine and cytokine receptor in the eutopic group. Among the pathway differences between overall normal endometrium and eutopic endometrium (Fig. 7F), we observed that overactivation of the Hedgehog (Hh) signaling pathway in eutopic endometrium warrants attention. Hh overactivation can lead to abnormal cell proliferation and cancer (Haider et al. 2019, Herrera et al. 2023), possibly making it the most crucial mechanistic pathway for EMT in endometriosis. Additionally, we found that overactivation of the prolactin signaling pathway and the natriuretic peptide receptor 2 (NPR2) signaling pathway may promote the proliferation and migration of endometriotic cells, thereby exacerbating the condition of endometriosis. Overactivation of the prolactin signal is closely related to the occurrence and development of various tumors (e.g. breast, prostate, and liver), driving tumor growth by promoting cell proliferation and inhibiting apoptosis (Haider et al. 2019, Deng et al. 2024, Sang et al. 2020). Furthermore, C-type natriuretic peptide (CNP) activates NPR2 receptors, increasing intracellular cyclic guanosine monophosphate (cGMP) levels and promoting cell proliferation and migration (Galetaki and Dauber 2024). In conclusion, overactivation of these key pathways lays the groundwork for the development and progression of eutopic endometriosis.

## Discussion

The biomarkers identified in this study – HNMT, CCDC28A, MGRN1 and FADS1 – are all expressed in the same anatomical region of the endometrium, making a comparative analysis logical. Furthermore, these biomarkers were rigorously selected through three screening methods: transcriptomics, single-cell analysis and MR. The screening results of 26 other candidate biomarkers did not align across these methods, excluding them from further analysis. MR analysis suggested that, as the expression levels of *FADS1* and *MGRN1*increase, the risk of developing endometriosis rises. One study showed that knockdown of FADS1 inhibits the proliferation, migration, and invasion of cancer cells (Zhao et al. 2020). Coincidentally, our analyses suggested that the primary event underlying the development of endometriosis was the occurrence of EMT in the eutopic endometrium. The first line of evidence for this was the significant reduction in the epithelial cell marker CDH1 (Figs.5H and 7B). The second was that compared with normal endometrium, there was a decrease in epithelial cells and an increase in stromal cells in eutopic endometrium (Fig. 5B). Epithelial cells are connected to each other through various types of junctions, including adherens junctions, desmosomes, gap junctions, and tight junctions. In contrast, mesenchymal cells do not possess functional epithelial junctions (Yang et al. 2020. Downregulation of *CDH1* leads to a decrease in E-cadherin levels, which in turn weakens cell-to-cell adhesion and enhances cellular motility. This improved ability to move may be the most critical factor in the migration of endometrial cells. This suggests that FADS1 may have a close relationship with EMT and CHD1. Similarly, some studies have reported that knockout of *MGRN1*leads to an increase in E-cadherin levels, resulting in stronger cell adhesion (Cerdido et al. 2024). In this study, eutopic endometrium exhibited upregulated expression of*MGRN1* and downregulated expression of *CDH1*, the E-cadherin coding gene, compared with normal endometrium. Both observations point to a reduction in cell adhesion, which facilitates cell dissemination and migration. Therefore, MGRN1 should also be given significant attention in the context of endometriosis. There are no reports in the literature directly linking the other two biomarkers, HNMT and CCDC28A, to endometriosis. However, our MR analysis suggested that decreased expression levels of *HNMT* and *CCDC28A* were associated with an increased risk of developing endometriosis.

The clinical sample collection of normal endometrial tissues was obtained from patients with uterine prolapse who no longer intended to conceive. However, we were unable to obtain eutopic endometrial samples from patients with endometriosis because most of these patients are of reproductive age, for whom taking eutopic endometrial tissue for experimental purposes could cause significant harm. Therefore, in this study, we only collected normal endometrial tissues and clinical samples from chocolate cysts and lesions that had metastasized to the pelvic peritoneum. The qPCR analysis of these samples revealed dramatically decreased expression levels of *CDH1* and *KRT23* in ectopic group (Fig. 5D). Interpreting this phenomenon as evidence of EMT occurring in ectopic tissue may be logically unsound considering the natural distinctions in tissue and cell proportions between samples derived from different anatomical locations. To resolve this issue, we analyzed the GSE213216 dataset. By comparing tissues from the same anatomical location, we found that the trends in *CDH1* and *KRT23* expression between the normal and lesion groups were less pronounced (Fig. 6C). Furthermore, the trend in the proportion of stromal cells in the was the inverse of what would be expected if EMT were progressing (Fig. 6B). We attribute this discrepancy to differences in anatomical location rather than the occurrence of EMT. However, compared with normal endometrial tissue, EMT did indeed occur in the eutopic endometrium. We found that *CDH1*was predominantly expressed in ciliated epithelial cells. Additionally, some studies have reported that, in endometriosis, the number of ciliated epithelial cells decreases, and the frequency of ciliary beating is downregulated (Devesa-Peiro et al. 2020). However, this ciliary phenotype has not received widespread attention. Under normal circumstances, the movement of cilia helps to expel endometrial-like cells from the uterine cavity. When ciliary function is impaired, these cells can remain in the pelvic cavity, potentially implanting and growing in ectopic locations, ultimately leading to endometriosis. This may represent a novel pathogenic perspective of endometriosis. Considering this role of cilia in endometriosis, targeting ciliary function may have potential as a future research direction. By modulating ciliary function or repairing structural abnormalities of cilia, it may be possible to reduce the formation and spread of ectopic endometrial tissue. Furthermore, consider that cilia, as a characteristic feature of many epithelial cells, may be lost as epithelial cells transition into mesenchymal cells. EMT is typically accompanied by a loss of cell polarity and cell–cell junctions, making the loss of cilia a potential hallmark of the process (Lee and Gleeson 2010). Cilia sense external signals and activate internal signaling pathways, such as the Hh signaling pathway (He et al. 2017), which play crucial roles in the EMT process. The loss or dysfunction of cilia can lead to abnormal activation or inhibition of these signaling pathways, thereby affecting the occurrence of EMT. Finally, through cell communication analysis, we found that ciliated epithelial cells in eutopic tissues were closely associated with B cells, T cells, and NK cells (Fig.7D, E). Additionally, we observed an increase in NK cell content within the eutopic endometrium (Figure S1), indicating that damage to ciliated epithelial cells may trigger a series of changes in the immune microenvironment (Fig. 7G). Currently, research on the role of ciliary epithelial cell damage and immune cell dysregulation in endometriosis is still in its early stages. Dysfunction of ciliary epithelial cells may alter the local immune microenvironment and activate chronic inflammatory responses. The accumulation of immune cells and the release of inflammatory mediators may further promote the growth and invasion of ectopic endometrial tissue, leading to impaired expulsion of endometrial cells and consequently activating local immune responses. However, this response fails to effectively clear ectopic endometrial cells. Repairing ciliary function and restoring immune cell function may offer new insights for the clinical treatment of endometriosis. For example, targeting the regulation of ciliary function to enhance immune cell recognition and clearance of ectopic endometrial cells may become an effective therapeutic strategy for endometriosis.We anticipate that future research will focus on this area, which could provide a new perspective on the pathogenesis of endometriosis.

## Supplementary Information

Below is the link to the electronic supplementary material.

ESM 1 (DOCX 6.01 MB)

ESM 2 (XLSX 39.1 KB)

ESM 3 (CSV 7.30 MB)

ESM 4 (CSV 4.47 MB)

ESM 5 (CSV 1.08 MB)

ESM 6 (CSV 161 KB)

ESM 7 (CSV 3.35 MB)

ESM 8 (CSV 4.26 MB)

## Data Availability

This study analyzed publicly available data sets. This study used data from public GEO data portal website (https://www.ncbi.nlm.nih.gov/geo/). Login id: (GSE11691 GSE7307 GSE7305 GSE23339 GSE25628 GSE213216 GSE179640).

## References

[CR1] Anvari S, Vyhlidal CA, Dai H, Jones BL (2015) Genetic variation along the histamine pathway in children with allergic versus nonallergic asthma. Am J Respir Cell Mol Biol 53:802–809. 10.1165/rcmb.2014-0493OC 25909280 10.1165/rcmb.2014-0493OC PMC4742940

[CR2] Bae S-J, Jo Y, Cho MK, Jin J-S, Kim J-Y, Shim J, Kim YH, Park J-K, Ryu D, Lee HJ et al (2022) Identification and analysis of novel endometriosis biomarkers via integrative bioinformatics. Front Endocrinol (Lausanne) 13:942368. 10.3389/fendo.2022.942368 36339397 10.3389/fendo.2022.942368 PMC9630743

[CR3] Becht E, McInnes L, Healy J, Dutertre C-A, Kwok IWH, Ng LG, Ginhoux F, Newell EW (2018) Dimensionality reduction for visualizing single-cell data using UMAP. Nat Biotechnol. 10.1038/nbt.4314 30531897 10.1038/nbt.4314

[CR4] Birney E (2022) Mendelian randomization. Cold Spring Harb Perspect Med 12:a041302. 10.1101/cshperspect.a041302 34872952 10.1101/cshperspect.a041302 PMC9121891

[CR5] Cerdido S, Abrisqueta M, Sánchez-Beltrán J, Lambertos A, Castejón-Griñán M, Muñoz C, Olivares C, García-Borrón JC, Jiménez-Cervantes C, Herraiz C (2024) MGRN1 depletion promotes intercellular adhesion in melanoma by upregulation of E-cadherin and inhibition of CDC42. Cancer Lett 581:216484. 10.1016/j.canlet.2023.216484 38008393 10.1016/j.canlet.2023.216484

[CR6] Decourtye-Espiard L, Guilford P (2023) Hereditary diffuse gastric Cancer. Gastroenterology 164:719–735. 10.1053/j.gastro.2023.01.038 36740198 10.1053/j.gastro.2023.01.038

[CR7] Devesa-Peiro A, Sebastian-Leon P, Garcia-Garcia F, Arnau V, Aleman A, Pellicer A, Diaz-Gimeno P (2020) Uterine disorders affecting female fertility: what are the molecular functions altered in endometrium? Fertil Steril 113:1261–1274. 10.1016/j.fertnstert.2020.01.025 32482256 10.1016/j.fertnstert.2020.01.025

[CR8] Deng K, Li X, Liu Z, Su Y, Sun X, Wei W, Fan Y, Zhang Y, Wang F (2024) IGF2BP2 regulates the proliferation and migration of endometrial stromal cells through the PI3K/AKT/mTOR signaling pathway in Hu sheep. J Anim Sci 102:skae129. 10.1093/jas/skae129 38727196 10.1093/jas/skae129 PMC11151927

[CR9] Dongre A, Weinberg RA (2019) New insights into the mechanisms of epithelial-mesenchymal transition and implications for cancer. Nat Rev Mol Cell Biol 20:69–84. 10.1038/s41580-018-0080-4 30459476 10.1038/s41580-018-0080-4

[CR10] Dudbridge F (2021) Polygenic mendelian randomization. Cold Spring Harb Perspect Med 11:a039586. 10.1101/cshperspect.a039586 32229610 10.1101/cshperspect.a039586 PMC7849343

[CR11] Galetaki DM, Dauber A (2024) C-Type natriuretic peptide analogs - current and future therapeutic applications. Horm Res Paediatr. 10.1159/000537743 38330932 10.1159/000537743

[CR12] Genome-wide association meta-analysis identifies new endometriosis risk loci - PubMed. https://pubmed.ncbi.nlm.nih.gov/23104006/ [Accessed August 14, 2024] 10.1038/ng.2445 PMC3527416 23104006

[CR13] García-Martín E, García-Menaya J, Sánchez B, Martínez C, Rosendo R, Agúndez J (2007) a. G. Polymorphisms of histamine-metabolizing enzymes and clinical manifestations of asthma and allergic rhinitis. Clin Exp Allergy 37:1175–1182. 10.1111/j.1365-2222.2007.02769.x 17651147 10.1111/j.1365-2222.2007.02769.x

[CR14] Haider S, Gamperl M, Burkard TR, Kunihs V, Kaindl U, Junttila S, Fiala C, Schmidt K, Mendjan S, Knöfler M et al (2019) Estrogen Signaling drives ciliogenesis in human endometrial organoids. Endocrinology 160:2282–2297 31290979 10.1210/en.2019-00314

[CR15] He M, Agbu S, Anderson KV (2017) Microtubule Motors drive hedgehog signaling in primary cilia. Trends Cell Biol 27:110–125. 10.1016/j.tcb.2016.09.010 27765513 10.1016/j.tcb.2016.09.010 PMC5258846

[CR16] Herrera L, Martin-Inaraja M, Bengoetxea A, Vendrell A, Pérez-Fernández S, Eguizabal C, Matorras R (2023) Natural killer cell subsets in endometrial fluid: a pilot study of their association with the endometrial cycle and reproductive parameters. J Assist Reprod Genet 40:2241–2250. 10.1007/s10815-023-02862-4 37436645 10.1007/s10815-023-02862-4 PMC10440323

[CR17] Hosseini M, Hammami B, Kazemi M (2023) Identification of potential diagnostic biomarkers and therapeutic targets for endometriosis based on bioinformatics and machine learning analysis. J Assist Reprod Genet 40:2439–2451. 10.1007/s10815-023-02903-y 37555920 10.1007/s10815-023-02903-y PMC10504186

[CR18] Ji X, Huang C, Mao H, Zhang Z, Zhang X, Yue B, Li X, Wu Q (2022) Identification of immune- and autophagy-related genes and effective diagnostic biomarkers in endometriosis: a bioinformatics analysis. Ann Transl Med 10:1397. 10.21037/atm-22-5979 36660690 10.21037/atm-22-5979 PMC9843312

[CR19] Jiang H, Zhang X, Wu Y, Zhang B, Wei J, Li J, Huang Y, Chen L, He X (2022) Bioinformatics identification and validation of biomarkers and infiltrating immune cells in endometriosis. Front Immunol 13:944683. 10.3389/fimmu.2022.944683 36524127 10.3389/fimmu.2022.944683 PMC9745028

[CR20] Kiesel L, Sourouni M (2019) Diagnosis of endometriosis in the 21st century. Climacteric 22:296–302. 10.1080/13697137.2019.1578743 30905186 10.1080/13697137.2019.1578743

[CR21] Koninckx PR, Fernandes R, Ussia A, Schindler L, Wattiez A, Al-Suwaidi S, Amro B, Al-Maamari B, Hakim Z, Tahlak M (2021) Pathogenesis Based Diagnosis and Treatment of Endometriosis. Front Endocrinol (Lausanne) 12:745548. 10.3389/fendo.2021.745548 34899597 10.3389/fendo.2021.745548 PMC8656967

[CR22] Korsunsky I, Millard N, Fan J, Slowikowski K, Zhang F, Wei K, Baglaenko Y, Brenner M, Loh P-R, Raychaudhuri S (2019) Fast, sensitive and accurate integration of single-cell data with Harmony. Nat Methods 16:1289–1296. 10.1038/s41592-019-0619-0 31740819 10.1038/s41592-019-0619-0 PMC6884693

[CR23] Kusama K, Fukushima Y, Yoshida K, Sakakibara H, Tsubata N, Yoshie M, Kojima J, Nishi H, Tamura K (2021) Endometrial epithelial-mesenchymal transition (EMT) by menstruation-related inflammatory factors during hypoxia. Mol Hum Reprod 27:gaab036. 10.1093/molehr/gaab036 33983443 10.1093/molehr/gaab036

[CR24] Lamouille S, Xu J, Derynck R (2014) Molecular mechanisms of epithelial-mesenchymal transition. Nat Rev Mol Cell Biol 15:178–196. 10.1038/nrm3758 24556840 10.1038/nrm3758 PMC4240281

[CR25] Lee JH, Gleeson JG (2010) The role of primary cilia in neuronal function. Neurobiol Dis 38:167–172. 10.1016/j.nbd.2009.12.022 20097287 10.1016/j.nbd.2009.12.022 PMC2953617

[CR26] Lyons RA, Saridogan E, Djahanbakhch O (2006) The reproductive significance of human fallopian tube cilia. Hum Reprod Update 12:363–372. 10.1093/humupd/dml012 16565155 10.1093/humupd/dml012

[CR27] Lieberman P (2011) The basics of histamine biology. Ann Allergy Asthma Immunol 106:S2–5. 10.1016/j.anai.2010.08.005 21277530 10.1016/j.anai.2010.08.005

[CR28] Owusu-Akyaw A, Krishnamoorthy K, Goldsmith LT, Morelli SS (2019) The role of mesenchymal-epithelial transition in endometrial function. Hum Reprod Update 25:114–133. 10.1093/humupd/dmy035 30407544 10.1093/humupd/dmy035

[CR29] Omega-6 fatty acids and inflammation - PubMed. https://pubmed.ncbi.nlm.nih.gov/29610056/ [Accessed August 20, 2024]

[CR30] Rahmioglu N, Nyholt DR, Morris AP, Missmer SA, Montgomery GW, Zondervan KT (2014) Genetic variants underlying risk of endometriosis: insights from meta-analysis of eight genome-wide association and replication datasets. Hum Reprod Update 20:702–716. 10.1093/humupd/dmu015 24676469 10.1093/humupd/dmu015 PMC4132588

[CR31] Saha R, Pettersson HJ, Svedberg P, Olovsson M, Bergqvist A, Marions L, Tornvall P, Kuja-Halkola R (2015) Heritability of endometriosis. Fertil Steril 104:947–952. 10.1016/j.fertnstert.2015.06.035 26209831 10.1016/j.fertnstert.2015.06.035

[CR32] Sang Y, Li Y, Xu L, Li D, Du M (2020) Regulatory mechanisms of endometrial decidualization and pregnancy-related diseases. Acta Biochim Biophys Sin 52:105–115. 10.1093/abbs/gmz146 31854442 10.1093/abbs/gmz146

[CR33] Simpson JL, Elias S, Malinak LR, Buttram VC (1980) Heritable aspects of endometriosis. I. genetic studies. Am J Obstet Gynecol 137:327–331. 10.1016/0002-9378(80)90917-5 7377252 10.1016/0002-9378(80)90917-5

[CR34] Simopoulos AP (2002) The importance of the ratio of omega-6/omega-3 essential fatty acids. Biomed Pharmacother 56:365–379. 10.1016/s0753-3322(02)00253-6 12442909 10.1016/s0753-3322(02)00253-6

[CR35] Stehr AM, Wang G, Demmler R, Stemmler MP, Krug J, Tripal P, Schmid B, Geppert CI, Hartmann A, Muñoz LE et al (2022) Neutrophil extracellular traps drive epithelial-mesenchymal transition of human colon cancer. J Pathol 256:455–467. 10.1002/path.5860 34939675 10.1002/path.5860

[CR36] Taylor HS, Kotlyar AM, Flores VA (2021) Endometriosis is a chronic systemic disease: clinical challenges and novel innovations. Lancet 397:839–852. 10.1016/S0140-6736(21)00389-5 33640070 10.1016/S0140-6736(21)00389-5

[CR37] Traag VA, Waltman L, van Eck NJ (2019) From Louvain to Leiden: guaranteeing well-connected communities. Sci Rep 9:5233. 10.1038/s41598-019-41695- 30914743 10.1038/s41598-019-41695-z PMC6435756

[CR38] Upadhyay A, Amanullah A, Chhangani D, Mishra R, Prasad A, Mishra A (2016) Mahogunin Ring Finger-1 (MGRN1), a multifaceted ubiquitin ligase: recent unraveling of neurobiological mechanisms. Mol Neurobiol 53:4484–4496. 10.1007/s12035-015-9379-8 26255182 10.1007/s12035-015-9379-8

[CR39] Wang Z, Liu J, Li M, Lian L, Cui X, Ng T-W, Zhu M (2022) Integrated bioinformatics analysis uncovers characteristic genes and molecular subtyping system for endometriosis. Front Pharmacol 13:932526. 10.3389/fphar.2022.932526 36059959 10.3389/fphar.2022.932526 PMC9428290

[CR40] Wang J, Lu Y, Sun G, Fang Z, Xing Z, Nong W, Wei Y, Wang S, Shi G, Dong M et al (2023) Machine learning algorithms for a novel cuproptosis-related gene signature of diagnostic and immune infiltration in endometriosis. Sci Rep 13:21603. 10.1038/s41598-023-48990-w 38062233 10.1038/s41598-023-48990-w PMC10703883

[CR41] Westra H-J, Peters MJ, Esko T, Yaghootkar H, Schurmann C, Kettunen J, Christiansen MW, Fairfax BP, Schramm K, Powell JE et al (2013) Systematic identification of trans eQTLs as putative drivers of known disease associations. Nat Genet 45:1238–1243. 10.1038/ng.2756 24013639 10.1038/ng.2756 PMC3991562

[CR42] Yang J, Antin P, Berx G, Blanpain C, Brabletz T, Bronner M, Campbell K, Cano A, Casanova J, Christofori G et al (2020) Guidelines and definitions for research on epithelial-mesenchymal transition. Nat Rev Mol Cell Biol 21:341–352. 10.1038/s41580-020-0237-9 32300252 10.1038/s41580-020-0237-9 PMC7250738

[CR43] Zhou H, Zhang Z, Qu R, Zhu H, Luo Y, Li Q, Mu J, Yu R, Zeng Y, Chen B et al (2024) CCDC28A deficiency causes sperm head defects, reduced sperm motility and male infertility in mice. Cell Mol Life Sci 81:174. 10.1007/s00018-024-05184-5 38597936 10.1007/s00018-024-05184-5 PMC11006775

[CR44] Zhao R, Tian L, Zhao B, Sun Y, Cao J, Chen K, Li F, Li M, Shang D, Liu M (2020) FADS1 promotes the progression of laryngeal squamous cell carcinoma through activating AKT/mTOR signaling. Cell Death Dis 11:272. 10.1038/s41419-020-2457-5 32332698 10.1038/s41419-020-2457-5 PMC7181692

[CR45] Zhou C, Feng M, Chen Y, Lv S, Zhang Y, Chen J, Zhang R, Huang X (2023) Unraveling immunotherapeutic targets for endometriosis: a transcriptomic and single-cell analysis. Front Immunol 14:1288263. 10.3389/fimmu.2023.1288263 38035102 10.3389/fimmu.2023.1288263 PMC10687456

