# Supplementary material for: A new perspective on endometriosis: Integrating eQTL mendelian randomization with transcriptomics and single-cell data analyses
Source: Funct Integr Genomics. 2025 Mar 26;25(1):75. doi: 10.1007/s10142-025-01543-y (PMC11947010; doi:10.1007/s10142-025-01543-y)
Supplement: Supplementary file 1 — (DOCX 6.01 MB) [file 10142_2025_1543_MOESM1_ESM.docx]

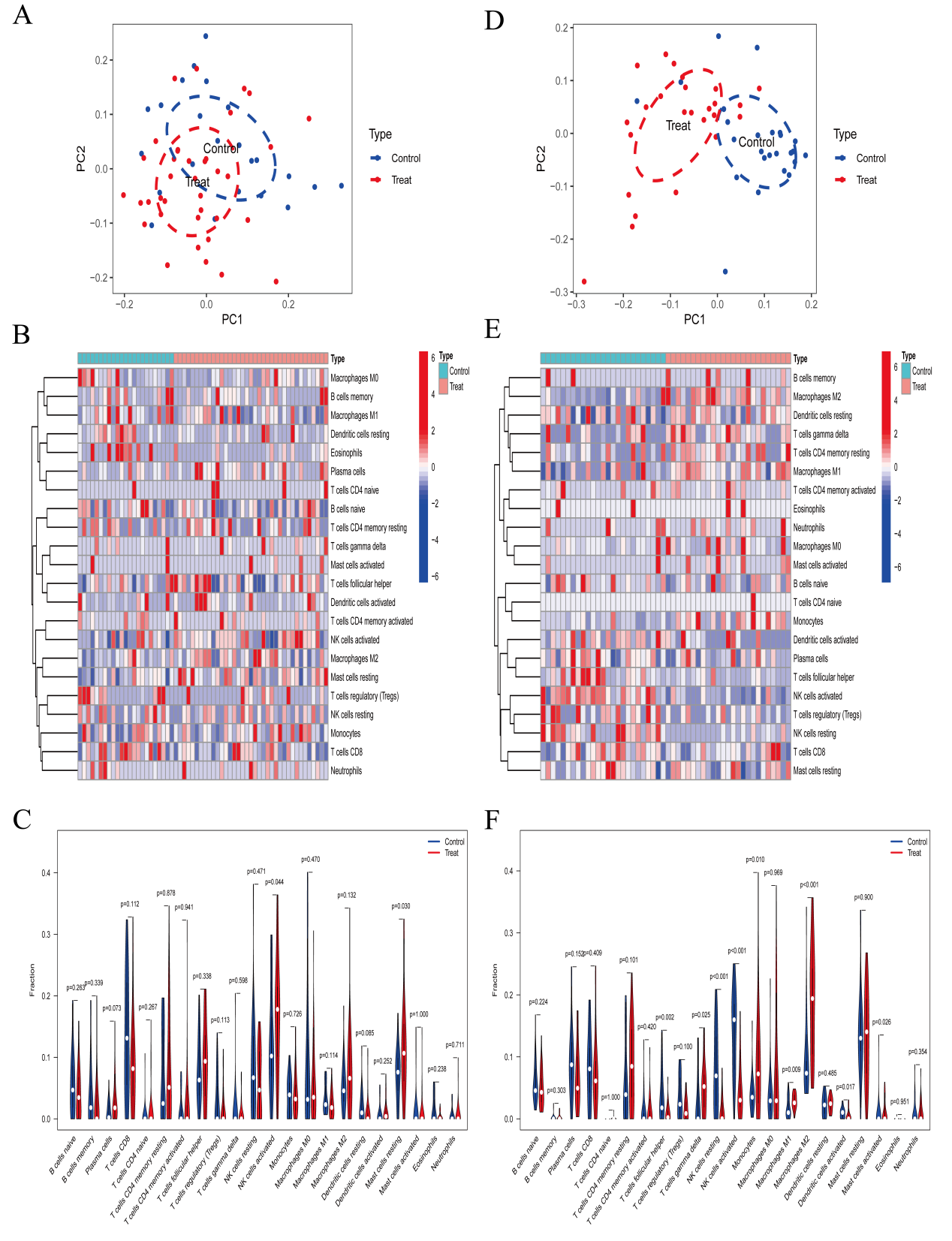
Supplementary Figure S1: A-C: Immune infiltration analysis in normal and eutopic samples. D-F: Immune infiltration analysis in eutopic and ectopic samples. A, D: Immune cell PCA analysis. B, E: Immune infiltration heatmap. C, F: The difference of immune cell content between the two groups was analyzed and the violin diagram of difference analysis was drawn.
